# Flow diverter-coil technique for endovascular treatment of complex wide neck brain aneurysms, Technical point

**DOI:** 10.22088/cjim.12.3.350

**Published:** 2021-04

**Authors:** Mohammad Ghorbani, Ebrahim Hejazian, Eshagh Bahrami, Abolghasem Mortazavi, Reza Bahrami, Nazila Farnoush

**Affiliations:** 1 Neuroendovascular Division, Firouzgar Hospital, Iran University of Medical Sciences (IUMS), Tehran, Iran; 2 Neuroendovascular Division, Ayatollah Rouhani Hospital, Babol University of Medical Sciences , Babol, Iran; 3 Department of neurosurgery, Rasool-e-Akram Hospital, Iran University of Medical Sciences, Tehran, Iran; 4 Division of Vascular and Endovascular Neurosurgery, Sina Hospital, Tehran University of Medical Sciences, Tehran, Iran; 5 Department of Neurosurgery, Tabriz University of Medical Sciences, Tabriz, Iran; 6 Department of Surgery, Babol University of Medical Sciences, Babol, Iran

**Keywords:** Complex wide neck brain aneurysms, Flow diverters, Coil, Treatment

## Abstract

**Background:**

Treatment of complex wide neck brain aneurysms is a challenging era in neurosurgery. Both surgical and endovascular therapies are considered for treatment of them. In endovascular, there are different ways such as trapping, coiling, stent and balloon assisted coiling. In this study, we use flow-diverter devices to create new vascular lumen and then coiling the aneurysm sac for three patients.

**Methods:**

We describe three cases with complex cerebral aneurysm who were treated successfully by flow diverter-coil technique and point to technical nuances.

**Results:**

In our patients, wide neck aneurysms, two in distal part of ICA (internal carotid artery) and other in basilar tip. We use flow-diverter-coil technique successfully. On the follow-up, aneurysms are treated completely without any complications.

**Conclusion:**

We think flow diverter devices adjunct to coiling is a useful way for the treatment of complex wide neck cerebral aneurysms.

Therapy of brain aneurysm dates back to 1937, when Dandy (1) described clipping of a posterior communicating artery aneurysm. For decades, clipping remained the gold standard treatment of cerebral aneurysms. Endovascular treatments emerged in the 1990s with the advent of the Guglielmi detachable coil system. This system established neurointervention as a new era, with multiple randomized clinical trials showing the efficacy and safety of coil embolization (2, 3). Despite these early good results, post coiling aneurysm recanalization remained a challenge. For example, Raymond et al. (4) experience with 501 cerebral aneurysms treated with coil embolization demonstrated that complete angiographic occlusion rate was about only 38% at 1-year follow-up. The data were further confirmed by Gory and Turjman,(5) whose prospective, multicenter European study of 404 aneurysms treated with Nexus detachable coils (ev3-Covidien, Irvine, California, USA), demonstrated 22% neck remnant and 30% aneurysmal remnant, with a 17.7% recanalization rate and 21.6% thrombosis rate at 13 months follow-up. In solving these problems, the neuroendovascular space quickly experienced very technological improvements in different properties of a coil, including coil lengths, shapes, softness and detachment zones. These developments improved clinical outcomes of cerebral aneurysms treated with this way. For example, HydroCoils (MicroVention, Tustin, California, USA) allowed treatment of more complex aneurysmal shapes and reduced recurrence rates compared with bare-platinum coils (6,7).

Additionally, new neuroendovascular instruments such as intracranial stents and balloons were developed to reinforced coil embolization. Stent-assisted coiling was developed to improve the occlusion rate and coil packing density of wide-necked large and giant aneurysms. In this procedure, the stent (similar to a balloon) is putting across the aneurysm neck, providing a framework to protect the parent artery. This decreases coil loop prolapse and allows for higher density coil packing, leading to a reduction in recurrence rates and higher rates of angiographic occlusion (8–10).

Despite these technological advancements , aneurysms with large diameters (>10 mm), wide necks, unfavorable dome-to-neck ratios (<2) and fusiform shapes remain therapeutic challenges, with as high as >20% poor outcome (aneurysm recurrence or treatment-related morbidity/ mortality) associated with treatment of large/giant aneurysms (11,12). To address these drawbacks, device innovations, including flow diversion was introduced.

Flow diversion is based on two concepts: (1) the placement of a high-mesh density stent in the parent vessel distorts blood flow to the aneurysm and (2) the stent provides a frame for which endothelium can grow, and isolating the aneurysm from the parent artery (13). Flow diversion allows for progressive intra-aneurysmal thrombosis over time with subsequent radiographic occlusion of the aneurysm. The advantage of endoluminal flow diversion over endovascular coiling is treatment of the weak abnormal arterial wall by providing a scaffold for neo-endothelialization to occur. This neo-endothelialization results in a timeable occlusion of the aneurysm and usually provides a curative outcome compared with the known recurrence associated with coiling.

The first introduction of flow diverter stent was in 2007, with the invention of the Pipeline Embolization Device (PED; Medtronic Neurovascular, Irvine, California, USA) (14).   In the USA, however, PED is the only available flow diverter after its approval by the Food and Drug Administration (FDA) in 2011 (15).

## Cases

In our three cases, 600 mg clopidogrel and also 650 mg aspirin were prescribed then a 50-60 IU/kg bolus of heparin was injected intravenously during the procedure. All procedures were done under general anesthesia. Patients went home on clopidogrel 75 mg daily and aspirin 325 mg daily for at least 3 months. Patient characteristics present in table 1.

**Table 1:** Patient characteristics

**Age, Sex**	**Clinical presentation**	**Past medical** **history**	**Angiographic findings**	**Follow up** **length**	**Follow up angiographic results**
23, F	Frontal headache	Migraine headache	Wide neck aneurysm on left ICA bifurcation	18 months	Complete treatment of aneurysm
25, M	Seizure	Seizure in childhood	Wide neck aneurysm on basilar tip-right P1 junction	3 months	Complete treatment of aneurysm
56, F	Headache	negative	Wide neck aneurysm of Distal of left ICA	1months	-

**Case 1**

**Figure 1:**
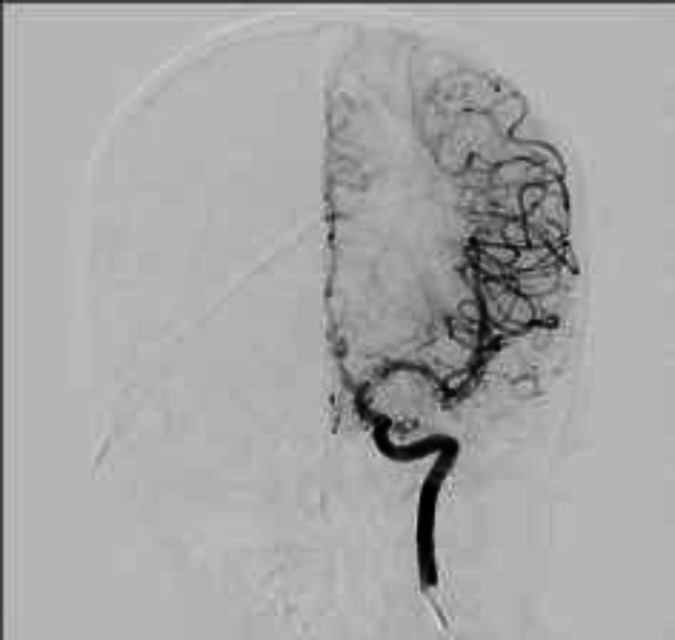
Wide neck aneurysm at ICA bifurcation

**Figure 2:**
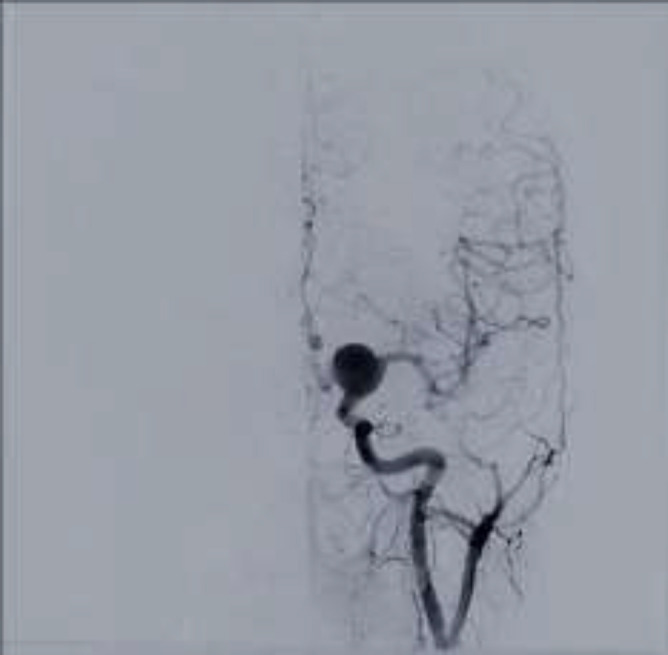
After 18 month angiography

Our first case is a-23-year old girl with chronic frontal headache and history of antimigraine drug use for pain. On evaluation, consisting of brain angiography, a wide neck saccular aneurysm at left internal carotid bifurcation was found (fig.1). Due to aneurysm configuration (existing both M1 and A1 arteries from the aneurysm sac) endovascular treatment only by coiling seemed to be dangerous. Therefore we put a flow-diverter stent in ICA-M1 to create new and safe lumen for parent artery, then from the right side of A-COM artery, we filled the aneurysm sac with coil. Before the procedure, she used clopidgrel and during that we administered heparin. The patient was discharged on clopidogrel and got well and the aneurysm was completely treated on angiogram (fig.2).

**Case2**

**Figure 3:**
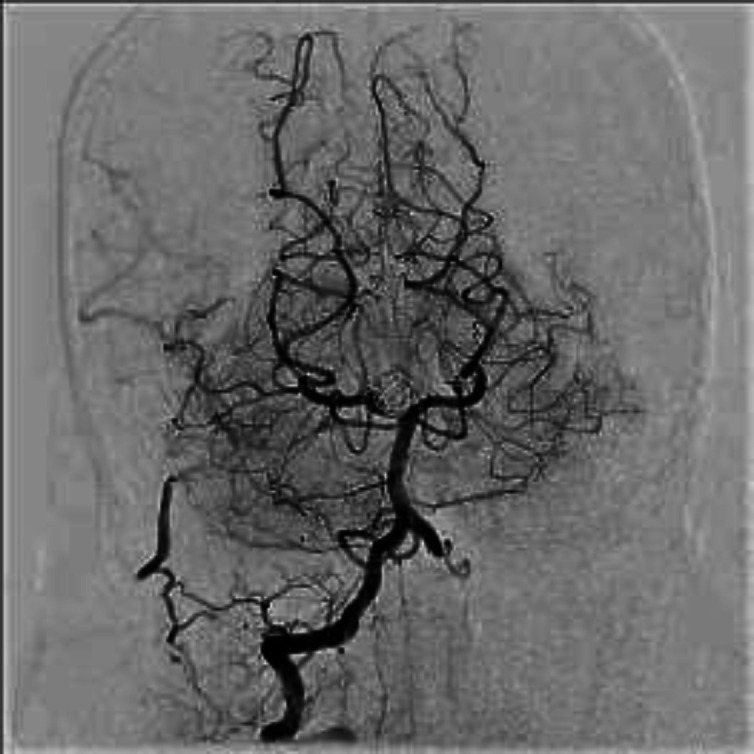
Basilar-P1 aneurysm

**Figure 4:**
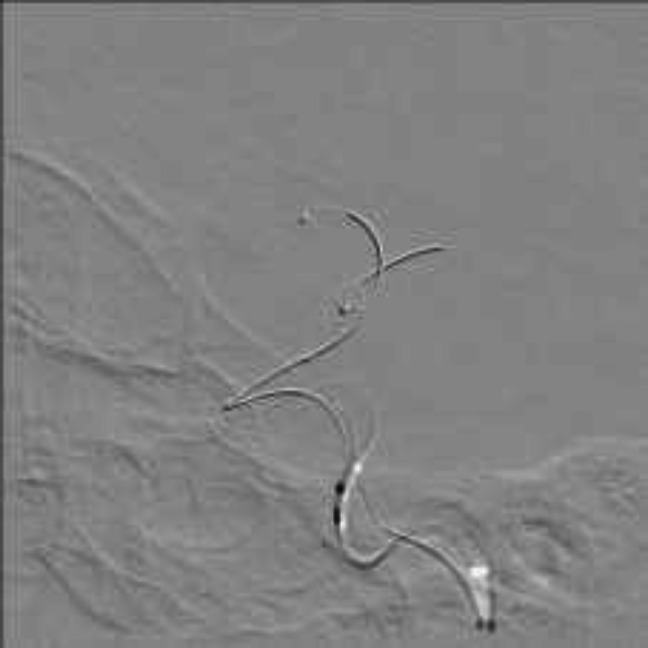
complete treated aneurysm

The second case is a 25-year-old boy afflicted to one episode of recent seizure. He had a history of cigarette smoking and two episodes of seizure on childhood. He was well on admission in our center. On evaluation, a wide neck saccular aneurysm was found at basilar artery- right P1 junction (fig 3). Similarly, due to aneurysm situation (right P1 originate from aneurysm sac) coiling alone is a dangerous option. Therefore, we put a fellow-diverter stent in basilar-right P1 to create new and safe lumen and then through previously positioned microcatheter in the aneurysm sac, coil placement in the sac was done (stent-assisted coiling). Before the procedure, he used same drugs same as the first case. After 2 months follow-up, he has had good condition and the aneurysm was completely treated angiographically (fig.4).

**Case 3**

**Figure 5:**
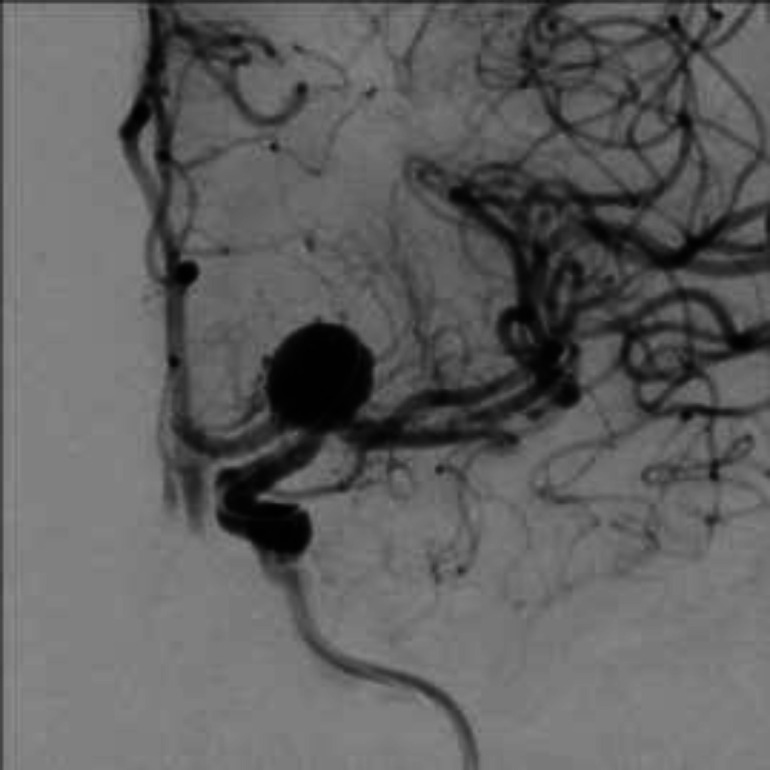
Left ICA distal wide neck aneurysm

**Figure 6:**
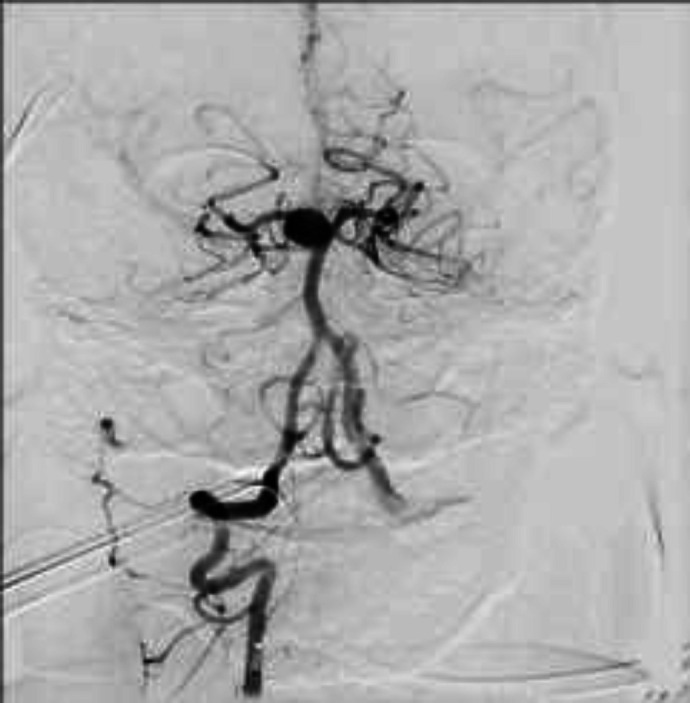
Microcatheterization and stenting

**Figure 7:**
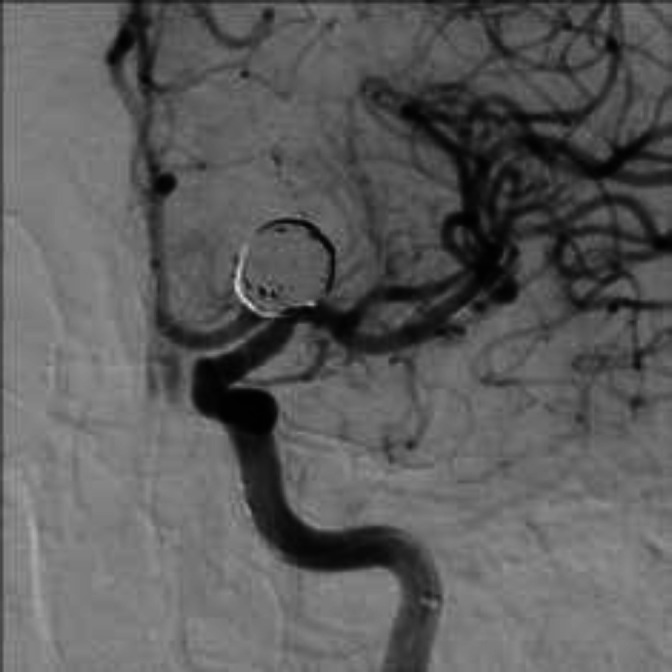
Complete treatment of aneurysm

The third case is a middle-aged woman evaluated for headache and her aneurysm was found incidentally. We decided to treat the aneurysm endovascularly despite the wide neck of it. Due to aneurysm configuration (existing both M1 and A1 arteries from the aneurysm sac, fig. 5) endovascular treatment only by coiling seemed to be dangerous. Therefore, we put a flow-diverter stent in ICA-M1 to create new and safe lumen for parent artery, then through the previously positioned microcatheter in the aneurysm sac through the left ICA, we filled the aneurysm sac with coil (figs 6, 7). Before the procedure, we administered same drugs as previous cases.She was well during the hospital stay and then went home on clopidogrel 75 mg and aspirin 325mg per day. The patient did not have a long-term follow-up.

## Discussion

Flow diverters are stent that are used endovascularly to treat cerebral aneurysms. Conceptually, flow diverters induced endoluminal reconstruction rather than endosaccular filling. The advantage is changing the parent artery/aneurysm sac interface, for example, altering in-flow and out-flow jets, to create aneurysm thrombosis. Intraaneurysmal thrombosis occur after device deployment. Subsequent neointimal growth covers the stent reconstructing the parent artery and thus, eliminate the aneurysm/parent vessel interface. This process usually spares the origins of perforating arteries (16, 17). These allow for timable reduction in rupture rates. Over time, the aneurysm sac shrinks and collapses and improving symptoms of mass effect (18). The thrombosis and associated inflammation of the aneurysm may induced temporary perianeurysmal edema in surrounding brain parenchyma (19). As compared to coil techniques, flow diverter devices cause aneurysms to obliterate over time rather than immediately at the end of the procedure. Therefore, aneurysm occlusion rates continue to increase between 6 and 12 months with flow diverters (20, 21). Perforators such as those from the middle cerebral artery or those from the basilar artery usually remain patent; however, obstructions may occur (17, 22). Alternatively, stent-assisted coiling was introduced, initially for wide-neck aneurysms, based on the hypothesis that a stent can provide the framework to hold the coils in the aneurysmal cavity (23, 24, 25).The use of antiplatelet drugs such as aspirin and clopidogrel is needed to prevent intraluminal stent thrombosis and stroke (26). David Dornbos reported endovascular treatment of small ruptured aneurysm of ophthalmic segment with flow diverter assisted coil to prevent coil migration (27).

In our patients, due to unfavorable configuration of aneurysms (wide neck or main artery originate from the aneurysm sac) we decided to use flow- diverter stents for create new and safe lumens for parent arteries and also as scaffold to hold the coils in the aneurysm sacs. Fortunately the aneurysms were treated completely and we did not face to any complications. Using antiplatelet drugs before stent and anticoagulant therapy during the procedure is very important to prevent thromboembolic events. And continuing antiplatelet therapy should be done for at least 3 months (maybe better for 6 months) after this treatment. 

In conclusion we think applying flow-diverter stents to creating new lumens for parent artery adjunct to endosaccular coiling is an appropriate way to treat complex wide neck cerebral aneurysms. Although more cases and studies are required to confirm this thesis. 

## Conflict of Interest statement:

The authors declare that the article content was composed in the absence of any commercial or financial relationships that could be construed as a potential conflict of interest.
